# Amplification on Undirected Population Structures: Comets Beat Stars

**DOI:** 10.1038/s41598-017-00107-w

**Published:** 2017-03-06

**Authors:** Andreas Pavlogiannis, Josef Tkadlec, Krishnendu Chatterjee, Martin A. Nowak

**Affiliations:** 1 IST Austria, Klosterneuburg, A-3400 Austria; 2 Program for Evolutionary Dynamics, Department of Organismic and Evolutionary Biology, Department of Mathematics, Harvard University, Cambridge, MA 02138 USA

## Abstract

The fixation probability is the probability that a new mutant introduced in a homogeneous population eventually takes over the entire population. The fixation probability is a fundamental quantity of natural selection, and known to depend on the population structure. Amplifiers of natural selection are population structures which increase the fixation probability of advantageous mutants, as compared to the baseline case of well-mixed populations. In this work we focus on symmetric population structures represented as undirected graphs. In the regime of undirected graphs, the strongest amplifier known has been the Star graph, and the existence of undirected graphs with stronger amplification properties has remained open for over a decade. In this work we present the *Comet* and *Comet-swarm* families of undirected graphs. We show that for a range of fitness values of the mutants, the Comet and Comet-swarm graphs have fixation probability strictly larger than the fixation probability of the Star graph, for fixed population size and at the limit of large populations, respectively.

## Introduction

Evolutionary dynamics study populations of reproducing individuals and the composition of the population over the course of time. A fundamental quantity is the *fixation probability* ^1–10^, which characterizes the chances of an invading mutant to get fixed in a homogeneous population of residents. The most well-known mathematical model for studying evolutionary dynamics on finite populations is the birth-death Moran process^11^. Initially, a population of *N* individuals consists of two types: *N* − 1 residents, and 1 invading mutant. The residents are associated with a normalized fitness of 1, whereas the invading mutant has a fitness advantage *r* > 1, which is constant and independent of the composition of the population. The population size remains fixed over the course of time. At each time point, an individual is chosen for reproduction with probability proportional to its fitness, and its offspring replaces an individual chosen uniformly at random. In this setting, the population is *well-mixed*, as the reproducing individual may replace any other individual. The *fixation probability* is defined as the probability that the Moran process results in a population of *N* mutants (i.e., the mutants get *fixed* in the population). The fixation probability, *ρ*, for well-mixed populations is a function of *r* and *N*, and equals

$$\rho (r,N)=\frac{1-{r}^{-1}}{1-{r}^{-N}}\mathrm{.}$$

It is well-known that population structure affects the evolutionary dynamics^5, 12–24^. Evolutionary graph theory models the population structure as a graph, where each vertex of the graph is occupied by one individual^3, 5, 25^. The edges of each vertex define the neighboring sites of that vertex in space. The *generalized* Moran process on a graph is identical to the Moran process on well-mixed populations, with the exception that each offspring can only replace a neighbor of the reproducing individual. The well-mixed population then follows as a special case of the generalized Moran process, where the individuals are spread on the vertices of a Clique (or complete graph) *K*_*N*_. A graph of *N* vertices *G*_*N*_ is said to *amplify selection* ^5^, if the fixation probability *ρ*(*r*, *G*_*N*_) of a randomly placed initial mutant on *G*_*N*_ is larger than the fixation probability on a well-mixed population of the same size (i.e., if *ρ*(*r*, *G*_*N*_) > *ρ*(*r*, *K*_*N*_)). The emerging question is then to what extent the population structure can amplify the fixation probability^5, 20, 21, 26, 27^.

In this work, we focus on the most commonly studied case, where the population structure is modeled as an undirected graph, and the initial mutant arises with uniform probability on each vertex. Ever since the landmark work of ref. 5, there has been immense interest in identifying selection amplifiers in this regime^15, 16, 26, 28–30^. Due to its combinatorial nature, the focus of such work has been primarily on simple structures with high degree of symmetry (e.g., Paths, Stars and Cycles). The intricacy of the problem has also given rise to computational approaches^31–37^, which rely on numerical calculations and Monte Carlo simulations to search for amplifiers among small graphs.

One graph that has attracted considerable attention is the *Star graph S*_*N*_, with fixation probability approximated by

$$\rho (r,{S}_{N})\simeq \frac{1-{r}^{-2}}{1-{r}^{-2N}}$$

for large enough *N*. More precise formulas have been derived in various works^15, 30, 38^. As *N* → ∞, the fixation probability on the Star becomes *ρ*(*r*, *S*_∞_) = 1 − *r* ^−2^. In contrast, the corresponding probability for the well-mixed population is *ρ*(*r*, *K*_∞_) = 1 − *r* ^−1^. Hence, the Star is a *quadratic amplifier*, as it effectively amplifies the selective advantage of mutants from *r* to *r* ^2^, where the well-mixed population is used as the basis of comparison. For over a decade of active study, the Star graph has been the strongest amplifier known for undirected graphs in the limit of large populations (as *N* → ∞). While for directed graphs stronger amplifiers are known (such as the Superstar^5^), the absence of stronger undirected amplifiers as compared to the Star graph has led to the conjecture that in the limit of large populations, among undirected graphs the Star graph is the strongest amplifier. The conjecture can be formalized as follows:

**Conjecture 1.** *For all values of r* ≥ 1, *for every infinite family of undirected graphs* (*G*_*N*_)_*N*≥1_, *we have* $\mathrm{lim}\,{{\rm{\inf }}}_{N\to \infty }\rho (r,{G}_{N})\le \,\mathrm{lim}\,{{\rm{\inf }}}_{N\to \infty }\rho (r,{S}_{N})$.

In the case of finite populations, exhaustive numerical calculations have revealed that there exist graphs of 9 vertices which for some values of *r* yield higher fixation probability that the fixation probability on *S*_9_^26^. However, amplification stronger than that on the Star graph has remained rare even in the case of finite populations.

In this work we present graphs with higher fixation probability than that on the Star graph, both for finite populations and at the limit of large populations, for some values of *r*. First, we present a graph *G*_*N*_ for a fixed size *N* which we call a *Comet*, and show that there exist values of *r* > 1 such that *ρ*(*r*, *G*_*N*_) > *ρ*(*r*, *S*_*N*_). Second, we present a family of graphs (*M*_*N*_)_*N*≥1_ and show that there exist values of *r* > 1 such that ${\mathrm{lim}}_{N\to \infty }\rho (r,{M}_{N}) > {\mathrm{lim}}_{N\to \infty }\rho (r,{S}_{N})$. This refutes Conjecture 1. The new graph family is called the *Metastar* family, which is a simple and natural extension of the Star family by replacing every leaf node with a graph *G*_*m*_ of small size. Our main result gives the fixation probability on Metastars as a function of the fixation probability on the small graph *G*_*m*_. We show that the Metastar family instantiated with Comet graphs as *G*_*m*_ leads to the refutation of the conjecture. Hence the counterexample is a simple and natural extension of the Star family.

## The Generalized Moran Process

We denote by *G*_*N*_ = (*V*_*N*_, *E*_*N*_) an undirected graph of *N* vertices, which is connected. Given a vertex *u* ∈ *V*_*N*_, we denote by Nh(*u*) the set of *neighbors* of *u*, i.e., the vertices *v* ∈ *V*_*N*_ such that (*u*, *v*) ∈ *E*_*N*_. The *degree* of *u* is the number of neighbors of *u*, i.e., deg(*u*) = |Nh(*u*)|. A population of *N* individuals is spread on the vertices of *G*_*N*_. Each individual is either a *resident* or a *mutant*. Mutants are associated with a *fitness advantage r* ≥ 1, whereas the fitness of residents is normalized to 1. A *configuration* X ⊆ *V*_*N*_ of *G*_*N*_ is the set of vertices of *G*_*N*_ that are occupied by mutants. The generalized Moran process on *G*_*N*_ is a discrete-time random process. Given a configuration X_*i*_ at time *i*, the next configuration at time *i* + 1 is determined by the following two events in succession.

1. One individual is chosen at random to reproduce, with probability proportional to its fitness. That is, the probability to reproduce is *r*/F(X_*i*_) for a mutant, and 1/F(X_*i*_) for a resident, where
  $${\rm{F}}({{\rm{X}}}_{i})=r\cdot |{{\rm{X}}}_{i}|+N-|{{\rm{X}}}_{i}|$$
  is the total population fitness. Let *u* be the vertex occupied by the reproducing individual.
2. A neighbor *v* ∈ Nh(*u*) is chosen uniformly at random. The individual occupying *v* dies, and the offspring of the reproducing individual is placed on *v*.

The mutants *reach fixation* in *G*_*N*_ if at some time point *i* we reach X_*i*_ = *V*, i.e., all vertices of *G*_*N*_ are occupied by mutants. The mutants *reach extinction* if at some time point *i* we reach X_*i*_ = ∅, i.e., all vertices of *G*_*N*_ are occupied by residents. We denote by *ρ*(*r*, *G*_*N*_) the probability that the mutants reach fixation in the generalized Moran process starting with a single, uniformly placed mutant on *G*_*N*_.

### The Clique and Star graphs

The Clique graph *K*_*N*_ consists of *N* vertices and an edge between each pair of vertices. The Star graph *S*_*N*_ consists of a single *root* vertex and *N* − 1 leaf vertices, and an edge between the root and each of the leaves. It is known that^3^

$$\rho (r,{K}_{N})=\frac{1-{r}^{-1}}{1-{r}^{-N}}\quad {\rm{and}}\quad \rho (r,{S}_{N})\simeq \frac{1-{r}^{-2}}{1-{r}^{-2N}}\mathrm{.}$$

In particular, the exact fixation probability on *S*_*N*_ is given by ref. 30

$$\rho (r,{S}_{N})=\frac{N-1}{N}\cdot \frac{{h}_{l}-1}{{h}_{c}\cdot {h}_{l}^{N-1}-1}+\frac{1}{N}\cdot \frac{{h}_{c}-1}{{h}_{c}\cdot {h}_{l}^{N-1}-1}$$

where

$${h}_{c}=\frac{r\cdot (N-\mathrm{1)}+1}{r\cdot (N-\mathrm{1)}+{r}^{2}}\quad {\rm{and}}\quad {h}_{l}=\frac{N-1+r}{{r}^{2}(N-\mathrm{1)}+r}\mathrm{.}$$

## The Comet Family of Amplifiers

In this section we introduce a new graph family called the *Comet* graph, and show that for some fixed population sizes and values of *r*, Comets amplify selection more strongly than Stars.

### The Comet graph ${C}_{N}^{m}$

**Figure 1:**
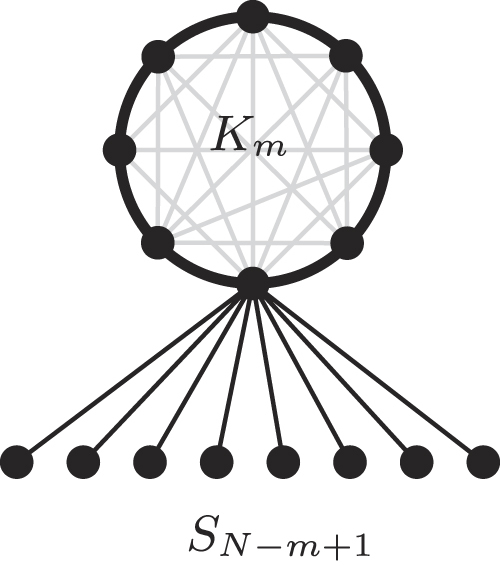
The Comet graph ${{C}}_{{N}}^{{m}}$ consists of a Clique *K*_*m*_ and a Star *S*_*N*−*m*+1_ graph.

Let *m* be any integer with 1 ≤ *m* ≤ *N*. The Comet graph ${C}_{N}^{m}$ consists of a Clique *K*_*m*_ of *m* vertices, where one vertex of the Clique is the root of a Star *S*_*N*−*m*+1_ of *N* − *m* + 1 vertices. Figure 1 shows an illustration. We refer to the Clique-part and the leaves of the Star-part of ${C}_{N}^{m}$ as the *head* and the *tail* of the Comet, respectively. Observe how the Clique and Star graphs of *N* vertices are a special case of the Comet graph, without a tail (i.e., ${K}_{N}={C}_{N}^{N}$) and the largest possible tail 1 (i.e., ${S}_{N}={C}_{N}^{1}$), respectively.

### Amplification on Comet graphs

**Figure 2:**
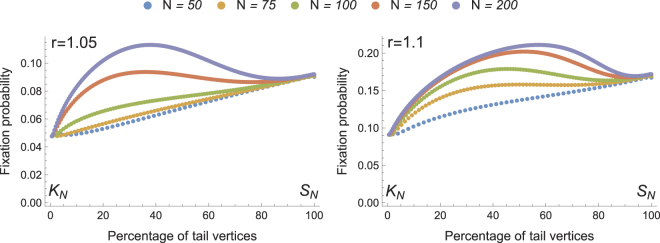
Fixation probabilities on the Comet graphs ${{C}}_{{N}}^{{m}}$. The Clique graph *K*_*N*_ and the Star *S*_*N*_ graph appear in the leftmost and rightmost points respectively. The X-axis shows the percentage of the vertices that appear in the tail of the Comet, with the remaining vertices appearing in the head of the Comet. In each case, all data-points which appear higher that the rightmost point of the plot correspond to Comets which amplify selection more strongly than the Star.

The Comet ${C}_{N}^{m}$ has the interesting property that for some values of *m* and *r*, it amplifies selection more strongly that the Star graph. Figure 2 shows the fixation probabilities on Comet graphs produced by keeping the population size *N* fixed, and varying the portion of the vertices that appear in the tail of the Comet. Remarkably, there is a range of graphs in between the two endpoints which amplify selection more strongly than the Star. For instance, we have $\rho \mathrm{(1.05,}\,{C}_{200}^{120})\ge 0.113$ and *ρ*(1.05, *S*_200_) < 0.093 which serves as a witness for stronger amplification than that on the Star graph.

## The Metastar Family of Amplifiers

In this section we refute Conjecture 1 for the limit of large populations. We introduce the *Metastar* graph, and compute the fixation probability of new mutants arising uniformly at random. Intuitively, the Metastar is identical to the Star, where each leaf vertex is replaced by a graph of small size. We will afterwards show how the Metastar family can be instantiated with such small graphs to refute Conjecture 1. We start with defining a variant of the generalized Moran process, called the lazy generalized Moran process.

## The *v*-lazy generalized Moran Process

### The *v*-lazy generalized Moran process

Given a distinguished vertex *v* ∈ *V*_*N*_, the *v-lazy* generalized Moran process on *G*_*N*_ is identical to the generalized Moran process on *G*_*N*_, except for the following modification. Whenever the reproducing individual occupies *v*, a biased coin with probability of heads 1/(deg(*v*) + 1) is flipped, so that

1. If the coin comes up heads, the individual replaces itself (i.e., the population remains unchanged).
2. If the coin comes up tails, the individual replaces one of its neighbors, chosen uniformly at random, as in the generalized Moran process.

Intuitively, the vertex *v* is considered a neighbor to itself when it comes to replacing a neighboring individual.

### Fixation probabilities

We consider fixation probabilities in the *v*-lazy generalized Moran process under two particular scenarios: (i) the initial mutant is placed on a vertex chosen uniformly at random, and (ii) the initial mutant is placed on a specific vertex. To refer to such events, we rely on the following notation.

- *ρ*(*r*, *G*_*N*_, *v*) is the probability that the mutants reach fixation in the *v*-lazy generalized Moran process starting with a single, uniformly placed mutant on *G*_*N*_.
- *ρ* ^+^(*r*, *G*_*N*_, *v*) is the probability that the mutants reach fixation in the *v*-lazy generalized Moran process starting with a single mutant placed on *v*.
- *ρ* ^−^(*r*, *G*_*N*_, *v*) is the probability that the mutants reach extinction in the *v*-lazy generalized Moran process starting with a single resident placed on *v*.

## The Metastar Family

Let *G*_*m*_ = (*V*_*m*_, *E*_*m*_) be any fixed graph of *m* vertices, and distinguish some *v* ∈ *V*_*m*_ as the *attachment vertex* of *G*_*m*_. Given some $n\in {{\mathbb{N}}}^{+}$, we let *N*(*n*) = *n* · *m* + 1, and construct the Metastar graph ${ {\mathcal M} }_{N(n)}^{{G}_{m}}$ parameterized by *G*_*m*_ as follows.

1. We introduce *n* copies of *G*_*m*_, and a new *root vertex s*.
2. We add an edge between the attachment vertex *v* of each copy of *G*_*m*_ and the root vertex *s*.

**Figure 3:**
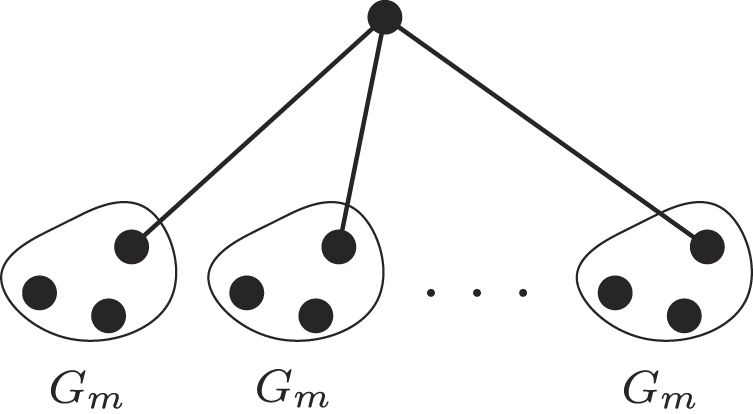
The Metastar graph ${ {\mathcal M} }_{{N}({n})}^{{{G}}_{{m}}}$ is identical to the Star graph, where every leaf is replaced by a small graph *G*_*m*_.

Figure 3 provides an illustration. From this point, we identify the *i*-th leaf of ${ {\mathcal M} }_{N(n)}^{{G}_{m}}$ with the *i*-th copy of *G*_*m*_.

## Fixation Probabilities on the Metastar

We now focus on the fixation probability on the Metastar. Since the graph is parameterized by *G*_*m*_, this probability depends on *G*_*m*_. However, because of the structure of ${ {\mathcal M} }_{N(n)}^{{G}_{m}}$, it does so in a modular way. This section outlines some key characteristics of the Moran process on Metastars, and presents intuitive arguments for the fixation probability. We refer to the Supplementary Information for the formal proofs. We first introduce some terminology which will help with the exposition of these ideas.

1. A leaf of ${ {\mathcal M} }_{N(n)}^{{G}_{m}}$ is called *heterogeneous* if mutants and residents coexist in that leaf, and *homogeneous* otherwise. A *mutant leaf* (resp. *resident leaf*) is a homogeneous leaf that contains only mutants (resp. residents).
2. We say that a leaf *i hits the root s* when the individual placed on the attachment vertex of the *i*-th copy of *G*_*m*_ places an offspring on *s*. Similarly, the root *s hits* leaf *i* when the individual placed on *s* places an offspring on the attachment vertex of the *i*-th copy of *G*_*m*_. We also say that a leaf *i hits another leaf j* at times (*t*_1_, *t*_2_) with *t*_1_ < *t*_2_ if leaf *i* hits the root at time *t*_1_ and the root hits leaf *j* at time *t*_2_, and the root is not hit again in the interval [*t*_1_, *t*_2_].

*Key idea*: The key idea in analyzing the fixation probability on ${ {\mathcal M} }_{N(n)}^{{G}_{m}}$ is to show that as *n* → ∞, every time the root hits a leaf *i*, or some leaf *i* hits another leaf *j*, the involved leaves are homogeneous with high probability. This is formally captured in the following two lemmas.

**Lemma 1.** *Consider that at some point the root hits a leaf i. The probability that the i-th leaf is heterogeneous the next time the root hits leaf i is* $O(1/\sqrt{n})$.

*Proof Idea.* Since the graph *G*_*m*_ of leaf *i* has constant size, the expected time for leaf *i* to reach a homogeneous state is *O*(*n*). On the other hand, the root *s* will need in expectation Ω(*n* ^2^) rounds to hit leaf *i*, as (i) *s* has *n* neighbors, and (ii) *s* reproduces approximately once every *N*(*n*) = Ω(*n*) rounds. The desired result then follows easily by applying concentration bounds. We refer to Lemma S1 in the Supplementary Information for the formal proof.□

Note that the complementary case of Lemma 1 does not hold, i.e., a heterogeneous leaf *i* will hit the root several times before leaf *i* becomes homogeneous. However, most of these events have no effect, as an offspring placed on the root by leaf *i* will be replaced by offsprings of other leaves, with high probability. The crucial event is the one in which a heterogeneous leaf *i* hits the root, and subsequently the root hits another leaf *j before* the root is hit again. Consider that leaf *i* becomes heterogeneous at some time *t*, and leaf *i* hits leaf *j* at times (*t*_1_, *t*_2_), with *t*_1_ > *t*. We call times (*t*, *t*_1_, *t*_2_) a *heterogeneous hit* if leaf *i* has remained heterogeneous throughout the interval [*t*, *t*_1_]. The following lemma states that heterogeneous hits are rare.

**Lemma 2.** *Consider that at some time t the i-th leaf is heterogeneous. The probability of a heterogeneous hit* (*t*, *t*_1_, *t*_2_) *is* $O(1/\sqrt{n})$.

*Proof Idea.* Note that in order for leaf *i* to hit leaf *j*, the following two events need to occur in succession.

(A) Leaf *i* hits the root *s*, and afterwards.
(B) The root *s* reproduces before it is hit.

First, we rely on Lemma 1 to conclude that with high probability, the root *s* does not hit leaf *i* before the latter becomes homogeneous. Hence, the probability that leaf *i* has remained heterogeneous in the interval [*t*, *t*_1_] is approximately the probability that the v-lazy generalized Moran process on *G*_*m*_ has not reached a homogeneous state.

Since *s* has *n* neighbors, the probability of event B happening in each round is *O*(1/*n*). Hence, in expectation, event *A* will need to happen Ω(*n*) times before leaf *i* hits leaf *j*. On the other hand, event *A* occurs with rate *O*(1/*n*). Thus the expected time required for leaf *i* to hit leaf *j* is Ω(*n* ^2^). Finally, since the graph *G*_*m*_ occupying leaf *i* has constant size, the expected time to reach a homogeneous state is only *O*(*n*). The desired result then follows easily by applying concentration bounds. We refer to Lemma S2 in the Supplementary Information for the formal proof.□

We are now ready to sketch the behavior of the Metastar. The initial mutant arises with high probability in one of the leaves, and is placed uniformly at random on one vertex of the corresponding graph *G*_*m*_. Lemma 1 implies that we can focus on that leaf in isolation. Since *v* is attached to the root *s*, the corresponding evolutionary process on *G*_*m*_ alone is the *v*-lazy generalized Moran process, and hence the invading mutant fixates in the initial leaf with probability *ρ*(*r*, *G*_*m*_, *v*). From that point on, Lemma 1 and Lemma 2 guarantee that the Metastar behaves like the Star, with the exception that

1. When the root hits a resident leaf with a mutant offspring, the leaf turns mutant with probability approximately *ρ* ^+^(*r*, *G*_*m*_, *v*).
2. When the root hits a mutant leaf with a resident offspring, the leaf turns resident with probability approximately *ρ* ^−^(*r*, *G*_*m*_, *v*).

In the case of the Star, both probabilities equal 1, since each leaf consists of a single vertex. Thus, if we focus on the ratio of probabilities of increasing the number of mutant leaves by one over decreasing it by one, this forward bias is amplified from *r* ^2^ (in the case of the Star) to *r* ^2^ · *ρ* ^+^(*r*, *G*_*m*_, *v*)/*ρ* ^−^(*r*, *G*_*m*_, *v*). We refer to the SI for the formal proof. The following theorem states the fixation probability on the Metastar.

**Theorem 1.** *Let G*_*m*_ *be a fixed graph and v the attachment vertex of G*_*m*_ *. Denote p = ρ(r, G*_*m*_ *, v) and α = ρ* ^−^(*r*, *G*_*m*_, *v*) *and β* = *ρ* ^+^(*r*, *G*_*m*_, *v*). *The fixation probability of a single mutant placed uniformly at random on* ${ {\mathcal M} }_{N(n)}^{{G}_{m}}$ *is*

1 $$\rho (r,{ {\mathcal M} }_{N(n)}^{{G}_{m}})\ge p\cdot \frac{1-{r}^{-2}\cdot (\alpha /\beta )}{1-{({r}^{-2}\cdot (\alpha /\beta ))}^{n}}\cdot \mathrm{(1}+o\mathrm{(1))}$$

Note that for the special case where *m* = 1 and *G*_*m*_ consists of a single vertex *G*_*m*_ = ({*v*}, ∅), we have *p* = *α* = *β* = 1, and eq. (1) gives the fixation probability on the Star graph. As *n* → ∞, we have *N* → ∞, and obtain that

2 $$\mathop{\mathrm{lim}}\limits_{N\to \infty }\rho (r,{ {\mathcal M} }_{N}^{{G}_{m}})\ge p\cdot \mathrm{(1}-{r}^{-2}\cdot (\alpha /\beta \mathrm{)).}$$

## Instances of the Metastar Family

In this section we present instances of the Metastar family. In particular we will instantiate the graphs *G*_*m*_ of the Metastar family with the Comet graphs of the previous Section.

### Metastar: The Comet-swarm ${{\boldsymbol{ {\mathcal M} }}}_{{\boldsymbol{N}}({\boldsymbol{n}})}^{{{\boldsymbol{C}}}_{{\bf{200}}}^{{\bf{100}}}}$

**Figure 4:**
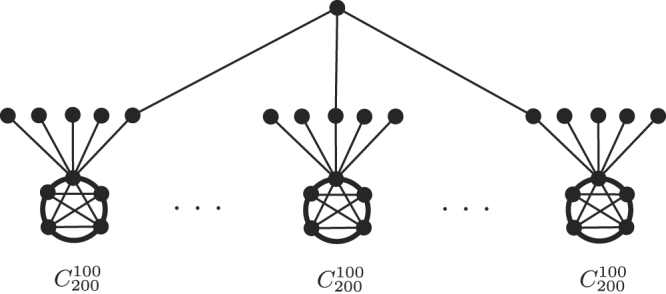
The Comet-swarm ${ {\mathcal M} }_{{N}}^{{{C}}_{200}^{100}}$.

We consider the Metastar ${ {\mathcal M} }_{N(n)}^{{C}_{200}^{100}}$ where each of the *n* leaves is a fixed-sized Comet ${C}_{200}^{100}$, and the attachment vertex *v* of ${C}_{200}^{100}$ is some arbitrary vertex of its tail (Fig. 4). We refer to this graph as the *Comet-swarm*, and obtain instances of various population sizes by increasing the number of leaves *n*.

**Figure 5:**
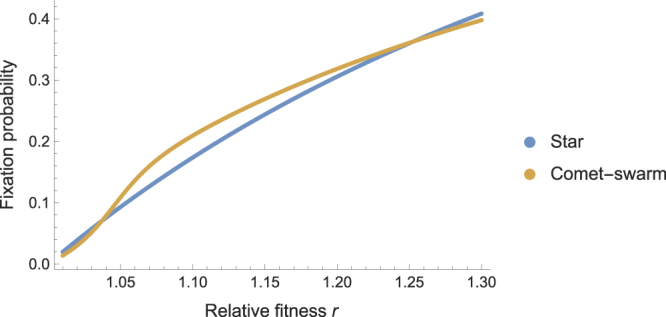
Fixation probabilities on ${ {\mathcal M} }_{{N}}^{{{C}}_{200}^{100}}$ for different values of relative fitness *r* and as *N* → ∞. For a range of values of *r* the Metastar amplifies more strongly than the Star.

As the size of ${C}_{200}^{100}$ is fixed, we can obtain the probabilities $p=\rho (r,{C}_{200}^{100},v)$ and $\alpha ={\rho }^{-}(r,{C}_{200}^{100},v)$ and $\beta ={\rho }^{+}(r,{C}_{200}^{100},v)$ for any *r*, by direct calculations. Figure 5 shows the fixation probability ${\mathrm{lim}}_{N\to \infty }\rho ({ {\mathcal M} }_{N}^{{C}_{200}^{100}})$ obtained from Eq. 2 for various values of *r*. In particular, we have

$$\mathop{\mathrm{lim}}\limits_{N\to \infty }\rho (\mathrm{1.1,}\,{ {\mathcal M} }_{N}^{{C}_{200}^{100}})=0.209\quad {\rm{and}}\quad \mathop{{\rm{l}}{\rm{i}}{\rm{m}}}\limits_{N\to \infty }\rho (\mathrm{1.12,}\,{S}_{N})=\mathrm{0.203;}$$

and thus obtain the following refutation of Conjecture 1 for the limit of large populations.

**Counterexample 1.** *For any r* ∈ [1.1, 1.12]*, we have that*

$$\mathop{\mathrm{lim}}\limits_{N\to \infty }\rho (r,{ {\mathcal M} }_{N}^{{C}_{200}^{100}}) > \mathop{\mathrm{lim}}\limits_{N\to \infty }\rho (r,{S}_{N}\mathrm{).}$$

## Discussion

The generalized Moran process studies the evolution of populations on spatial structures. To understand the impact of the underlying topology, efforts have focused on characterizing the extremes of this process, i.e., the maximum amplification of selection that can be attained. The combinatorial nature of the problem makes it difficult for mathematical analysis, and most works focus on either simple graphs or asymmetric topologies, represented as directed graphs^5^. Directed graphs can exhibit extreme behavior, from strongly amplifying selection (fixation with probability 1) to strongly suppressing it (fixation with probability 0). There even exist directed graphs where neither fixation nor extinction is possible. On the other hand, symmetric structures enjoy smoother behavior, as the population always resolves to a homogeneous state. In many cases symmetry is a very natural property, i.e., if an individual *A* can influence and individual *B*, then *B* can also influence *A*. Thus, amplification on undirected graphs is a very natural question to study.

It has been conjectured that the Star graph is the strongest amplifier of natural selection among undirected population structures. In this work we refute the conjecture both for fixed population sizes (with the Comet graph) and at the limit of large populations (with the Comet-swarm Metastar family of graphs), for a range of values of *r*. The Metastar family is a simple and natural extension of the well-studied Star family, where every leaf node is replaced with a graph of small size. We show that this simple and natural extension of Stars is sufficient for refuting the long-standing conjecture. Our results shed new light into the world of selection amplifiers, and we hope that they will inspire further research on this fascinating topic.

## Electronic supplementary material

Supplementary Information for
