# Supplementary material for: Amplification on Undirected Population Structures: Comets Beat Stars
Source: Sci Rep. 2017 Mar 6;7:82. doi: 10.1038/s41598-017-00107-w (PMC5427850; doi:10.1038/s41598-017-00107-w)
Supplement: Supplementary file 1 — Supplementary Information for [file 41598_2017_107_MOESM1_ESM.pdf]

Supplementary Information for "Amplification on Undirected Population Structures: Comets Beat Stars'

Andreas Pavlogiannis[1*†], Josef Tkadlec[1*],
Krishnendu Chatterjee[1], Martin A. Nowak[2]

[1]IST Austria, Klosterneuburg, A-3400, Austria
[2]Program for Evolutionary Dynamics, Department of Organismic and Evolutionary Biology,
Department of Mathematics, Harvard University, Cambridge, MA 02138, USA

[*]These authors contributed equally to this work.
[†]To whom correspondence should be addressed; E-mail: pavlogiannis@ist.ac.at.

In this document we introduce the Metastar family of graphs and establish formally the fixation probability of mutants arising uniformly at random on a Metastar. The Metastar family is parametric wrt a small graph of fixed size, and the fixation probability depends on this fixed graph. We refer to the main article for particular instances of the Metastar family with specific fixed graphs, which result in Metastar graphs that amplify selection more strongly than Star graphs, for various fitness values of the invading mutants.

1 Preliminaries

In this section we define formally the Moran Process on graphs, and introduce several definitions and notation that will help with the exposition of the ideas in this work.

1.1 The Moran Process on Structured Populations

We denote by $G_N = (V_N, E_N)$ an undirected graph of N vertices, which is connected. Given a vertex $u \in V_N$, we denote by $\mathsf{Nh}(u)$ the set of *neighbors* of u, i.e., the vertices $v \in V_N$ such that $(u, v) \in E_N$. The *degree* of u is the number of neighbors of u, i.e., $\mathsf{deg}(u) = |\mathsf{Nh}(u)|$. A population of N individuals is spread on the vertices of G_N. Each individual is either a *resident* or a *mutant*. Mutants are associated with a *fitness advantage* $r \geq 1$, whereas the fitness of residents is normalized to 1. A *configuration* $\mathsf{S} \subseteq V_N$ of G_N is the set of vertices of G_N that are occupied by mutants. The generalized Moran process on G_N is a discrete-time random process. Given a configuration S_i at time i, the next configuration at time $i + 1$ is determined by the following two events in succession.

1. One individual is chosen at random to reproduce, with probability proportional to its fitness. That is, the probability to reproduce is $r/\mathsf{F}(\mathsf{S}_i)$ for a mutant, and $1/\mathsf{F}(\mathsf{S}_i)$ for a resident, where

$$\mathsf{F}(\mathsf{S}_i) = r \cdot |\mathsf{S}_i| + N - |\mathsf{S}_i|$$

   is the total population fitness. Let u be the vertex occupied by the reproducing individual.
2. A neighbor $v \in \mathsf{Nh}(u)$ is chosen uniformly at random. The individual occupying v dies, and the offspring of the reproducing individual is placed on v.

The v-lazy Moran process. Given an undirected graph $G = (V, E)$ and a distinguished node $v \in V$, the *v-lazy* Moran process on G is the regular Moran process on G with the following modification. Whenever the reproducing individual is one that occupies the node v, a biased coin with probability of heads $1/(\mathtt{deg}(v) + 1)$ is flipped, so that

1. if the coin comes up heads, the individual replaces itself (i.e., the population remains unchanged);
2. if the coin comes up tails, the individual replaces one of its neighbors, chosen uniformly at random (as in the regular Moran process).

Intuitively, the node v is considered a neighbor of itself when it comes to replacing a neighboring individual. We will often call v the *lazy node* in this process.

1.2 Fixation Probabilities

The mutants *reach fixation* in G_N if at some time point i we reach $\mathsf{S}_i = V$, i.e., all vertices of G_N are occupied by mutants. The mutants *reach extinction* if at some time point i we reach $\mathsf{S}_i = \emptyset$, i.e., all vertices of G_N are occupied by residents. We denote by $\rho(r, G_N)$ the probability that the mutants reach fixation in the generalized Moran process starting with a single, uniformly placed mutant on G_N. Given a heterogeneous population (where mutants and residents coexist) spread out on a graph $G = (V, E)$ the Moran process on G almost surely reaches a state where the mutants either fixate in the population or go extinct. Our interest is on the probability that starting from a state where a single mutant coexists with $N - 1$ residents, the mutant eventually fixates. In general, this probability depends on the node that the mutant occupies initially. We consider two particular scenarios: (i) the initial mutant is placed on a node chosen uniformly at random, and (ii) the initial mutant is placed on a specific node. To refer to such events, we rely on the following notation.

- $\rho(r, G_N)$ is the probability that the mutants reach fixation in the generalized Moran process starting from a single, uniformly placed mutant on G_N ;
- $\rho(r, G_N, v)$ is the probability that the mutants reach fixation in the v-lazy generalized Moran process starting with a single, uniformly placed mutant on G_N;
- $\rho^{+}(r, G_N, v)$ is the probability that the mutants reach fixation in the v-lazy generalized Moran process starting with a single mutant placed on v;
- $\rho^{-}(r, G_N, v)$ is the probability that the mutants reach extinction in the v-lazy generalized Moran process starting with a single resident placed on v.

The Clique and Star graphs. The Clique graph K_N consists of N vertices and an edge between each pair of vertices. The Star graph S_N consists of a single *root* vertex and $N - 1$ leaf vertices, and an edge between the root and each of the leaves. It is known that [1]

$$\rho(r, K_N) = \frac{1 - r^{-1}}{1 - r^{-N}} \qquad \text{and} \qquad \rho(r, S_N) \simeq \frac{1 - r^{-2}}{1 - r^{-2N}}.$$

2 The Metastar Family of Selection Amplifiers

In this section we introduce the Metastar family of graphs, and prove a general theorem about the fixation probability of mutants on the Metastar. The family is parameterized by a small graph of fixed size and naturally, the fixation probability depends on this parameter. However, because of the structure of the Metastar, it does so in a modular way.

The Metastar family $\mathsf{M}^{G_m}_{N(n)}$. Let $G_m = (V_m, E_m)$ be any fixed graph of m vertices, and distinguish some $v \in V_m$ as the *attachment vertex* of G_m. Given some $n \in \mathbb{N}^{+}$, we let $N(n) = n \cdot m + 1$, and construct the Metastar graph $\mathsf{M}^{G_m}_{N(n)}$ parameterized by G_m as follows.

1. We introduce n copies of G_m, and a new *root vertex s*.
2. We add an edge between the attachment vertex v of each copy of G_m and the root vertex s.

and we identify the i-th leaf of $\mathsf{M}_{N(n)}^{G_m}$ with the i-th copy of G_m.

2.1 Random Trajectories and the Modified Moran Process on $\mathsf{M}_{N(n)}^{G_m}$

We first introduce some notation that will help with the exposition of the ideas in this section.

1. Given a configuration S, a leaf of $\mathsf{M}_{N(n)}^{G_m}$ is called *heterogeneous* in X if mutants and residents coexist in that leaf, and *homogeneous* otherwise. A *mutant leaf* (resp. *resident leaf*) is a homogeneous leaf that contains only mutants (resp. residents).
2. A *trajectory* ψ generated by the Moran process on $\mathsf{M}_{N(n)}^{G_m}$ is a sequence of events $\psi = e_0, e_1, \dots, e_k$, where e_0 is the vertex of $\mathsf{M}_{N(n)}^{G_m}$ that contains the initial mutant, and for $1 \leq i \leq k$, $e_i = (a_i, b_i)$ is a pair denoting the vertices a_i and b_i occupied by the reproducing and dying individuals respectively. Given some $i > 0$, we denote by ψ_i the prefix of ψ up to position $i-1$. We let $\mathcal{X}_i$ be a random variable that indicates the i-th event of ψ, and write $\mathbb{P}\left[\mathcal{X}_i = e_i | \psi_i\right]$ to denote the probability that $\mathcal{X}_i$ is realized to e_i given the trajectory ψ_i up to position $i-1$.
3. A *modified trajectory* π is obtained from a trajectory ψ by removing some events $e_i = (a_i, b_i)$ where $a_i = s$ is the root of $\mathsf{M}_{N(n)}^{G_m}$. To contrast with the modified trajectory π, we sometimes refer to ψ as a *regular* trajectory.
4. We say that a leaf i *hits the root* s at time t in a trajectory ψ if $e_t = (a_t, b_t)$ and a_t is the attachment vertex of the i-th copy of G_m, and b_t is the root s. Similarly, we say that the root s *hits* leaf i at time t in ψ if $e_t = (a_t, b_t)$ and a_t is the root s and b_t is the attachment vertex of the i-th copy of G_m. We also say that a leaf i *hits another leaf* j at times (t_1, t_2) with $t_1 < t_2$ if leaf i hits the root at time t_1 and the root hits leaf j at time t_2, and the root is not hit again in the interval $[t_1, t_2]$. In such a case, we call (t_1, t_2) a *hitting pair*. A *heterogeneous hit* from leaf i to leaf j occurs at times (t_1, t_2, t_3) if
   (a) The root hits leaf i at time t_1, and
   (b) leaf i hits leaf j at times (t_2, t_3), and
   (c) leaf i is heterogeneous in throughout the interval $(t_1, t_2]$.

The modified Moran process. The *modified* Moran process on $\mathsf{M}_{N(n)}^{G_m}$ consists of the regular Moran process with the following modifications:

1. if at any point the root hits a heterogeneous leaf i, then leaf i becomes instantaneously a resident leaf, and
2. if at any point a heterogeneous leaf i hits a leaf j, then leaf j becomes instantaneously a resident leaf.

Observe that every time Item 1 or Item 2 applies, the modified Moran process transitions to a configuration S' while the regular Moran process would transition to a configuration S and such that set of vertices occupied by mutants in X' is a subset of the set of vertices occupied by mutants in S. Thus the fixation probability from S is at least as large as the fixation probability from S'. We will use the modified Moran process on $\mathsf{M}_{N(n)}^{G_m}$ to underapproximate the fixation probability $\rho(r, \mathsf{M}_{N(n)}^{G_m})$.

2.2 The Interference of Heterogeneous Leaves

In this section we prove some useful lemmas regarding the modified Moran process on $\mathsf{M}_{N(n)}^{G_m}$. In particular, we show that every time the root hits a leaf i, or a leaf i hits another leaf j, the involved leaves can be considered homogeneous whp (Lemma S1 and Lemma S2). Additionally, we show that if the root reproduces in any two times t_1 and t_2, with $t_1 < t_2$, the root is hit in the interval (t_1, t_2) whp (Lemma S3). Finally, in Lemma S4 we characterize the probability that a hitting pair (t_1, t_2) is such that the individual reproducing in time t_1 comes from a mutant leaf, over the probability that this individual comes from a resident leaf.

The following lemma states that once a homogeneous leaf is hit by the root, whp that leaf will evolve independently of the root until it becomes homogeneous again.

Lemma S1. *Let π be a random modified trajectory, and consider that the root hits a leaf i at some time t. The probability that the i-th leaf is heterogeneous the next time it is hit by the root is $O(1/\sqrt{n})$.*

Proof Idea. Since the graph G_m of leaf i has constant size, the expected time for leaf i to reach a homogeneous state is $O(n)$. On the other hand, the root s will need in expectation $\Omega(n^2)$ rounds to hit leaf i, as (i) s has n neighbors, and (ii) s reproduces approximately once every $N(n) = \Omega(n)$ rounds. The desired result then follows easily by applying concentration bounds. □

Proof. Let $y = \sqrt{n}$. The event of leaf i being heterogeneous when hit by the root requires that either

(A) leaf i is hit by the root before y reproduction events have occurred locally in the vertices of leaf i, or

(B) leaf i has remained heterogeneous after y reproduction events have occurred locally.

We will show that both events happen with probability $O(1/\sqrt{n})$.

Let X be the random variable that indicates the number of rounds until the root hits leaf i for the first time after time t. Note that in a random modified trajectory the root reproduces with rate no larger than the rate with which it reproduces in a random regular trajectory. We lower-bound X by a random variable X' which realizes the same event while assuming that the root is always occupied by a mutant, and the rest of the population consists only of residents, and additionally π is a random regular trajectory. In particular, we have $\mathbb{P}[X \leq \alpha] \leq \mathbb{P}[X' \leq \alpha]$ for all α, where X' is geometrically distributed with rate r/n^2, which is an upper bound on the probability of the root reproducing and hitting leaf i, that is

$$X' \sim \mathsf{GM}(p_X) \qquad \text{where} \qquad p_X = \frac{r}{n^2}$$

Let Y_y be the random variable that denotes the number of rounds required for y reproduction events to take place in leaf i after time t. We upper-bound Y_y by another random variable Y_y' which realizes the same event while assuming that leaf i is a resident leaf, and the rest of the population contains only mutants. We have $\mathbb{P}[Y_y \geq \alpha] \leq \mathbb{P}\left[Y_y' \geq \alpha\right]$ for all α, with Y_y' drawn from the negative binomial distribution of receiving y failures with success rate equal to the probability of choosing an individual to reproduce that does not belong leaf I, that is

$$Y_y' \sim \mathsf{NB}(y, p_Y) \quad \text{where} \quad p_Y = \frac{((n-1)\cdot m + 1)\cdot r}{((n-1)\cdot m + 1)\cdot r + m} \qquad \mathsf{E}[Y_y'] = \frac{p_Y \cdot y}{1-p_Y} \qquad \mathsf{Var[Y_y']} = \frac{p_Y \cdot y}{(1-p_Y)^2} \tag{1}$$

Since the size of leaf i is constant (recall that $m = O(1)$), the expected number of local reproduction events that make leaf i homogeneous is constant (i.e., independent of n). By an easy application of Markov's inequality, event B happens with probability $O(1/\sqrt{n})$. On the other hand, event A happens with probability $\mathbb{P}[X < Y_y]$. Let $\alpha = c \cdot n^{3/2}$ where c is a sufficiently large constant, and note that

$$\mathbb{P}[X < Y_y] = \mathbb{P}[X < Y_y \leq \alpha] + \mathbb{P}[X < \alpha < Y_y] + \mathbb{P}[\alpha \leq X < Y_y] \leq \mathbb{P}\left[Y_y' \geq \alpha\right] + \mathbb{P}[X' \leq \alpha] \tag{2}$$

where $X' \sim \mathsf{GM}(p_X)$ and $Y_y' \sim \mathsf{NB}(y, p_Y)$ as defined above. We have

$$\mathbb{P}[X' \leq \alpha] = 1 - (1-p_X)^{\alpha} = 1 - \left(1 - \frac{r}{n^2}\right)^{c\cdot n^{3/2}} = O(1/\sqrt{n}) \tag{3}$$

and

$$\begin{aligned}\mathbb{P}\left[Y'_y \geq \alpha\right] &= \mathbb{P}\left[Y'_y - \mathsf{E}[Y'_y] \geq \alpha - \mathsf{E}[Y'_y]\right] \\ &\leq \mathbb{P}\left[|Y'_y - \mathsf{E}[Y'_y]| \geq \alpha - \mathsf{E}[Y'_y]\right] \\ &\leq \mathbb{P}\left[|Y'_y - \mathsf{E}[Y'_y]| \geq \frac{\alpha - \mathsf{E}[Y'_y]}{\sqrt{\mathsf{Var}[\mathsf{Y}'_\mathsf{y}]}} \cdot \sqrt{\mathsf{Var}[\mathsf{Y}'_\mathsf{y}]}\right] \\ &\leq \frac{\mathsf{Var}[\mathsf{Y}'_\mathsf{y}]}{\left(\alpha - \mathsf{E}[Y'_y]\right)^2} \end{aligned} \tag{4}$$

where the last inequality is obtained by applying Chebyshev's inequality (given by the formula $\mathbb{P}\left[|\mathcal{X} - \mathsf{E}[\mathcal{X}]| \geq k \cdot \sqrt{\mathsf{Var}[\mathcal{X}]}\right] \leq \frac{1}{k^2}$, where we substitute $k = \frac{\alpha - \mathsf{E}[Y'_y]}{\sqrt{\mathsf{Var}[\mathsf{Y}'_\mathsf{y}]}}$).

Note that since $m = O(1)$ and $r = O(1)$, by straightforward calculations for the variance in Eq. (1) we obtain

$$\mathsf{Var}[\mathsf{Y}'_\mathsf{y}] = \frac{p_Y \cdot y}{(1-p_Y)^2} = \frac{\frac{((n-1)\cdot m+1)\cdot r\cdot\sqrt{n}}{((n-1)\cdot m+1)\cdot r+m}}{\left(\frac{m}{((n-1)\cdot m+1)\cdot r+m}\right)^2} = O(n^{5/2}).$$

Similarly for the expected value, we obtain

$$\mathsf{E}[Y'_y] = \frac{p_Y \cdot y}{1-p_Y} = \frac{\frac{((n-1)\cdot m+1)\cdot r\cdot\sqrt{n}}{((n-1)\cdot m+1)\cdot r+m}}{\frac{m}{((n-1)\cdot m+1)\cdot r+m}} = O(n^{3/2})$$

and thus

$$(\alpha - \mathsf{E}[Y'_y])^2 = (c \cdot n^{3/2} - O(n^{3/2}))^2 = \Omega(n^3).$$

Substituting to Eq. (4), we obtain

$$\mathbb{P}\left[Y'_y \geq \alpha\right] =\leq \frac{O(n^{5/2})}{\Omega(n^3)} = O(1/\sqrt{n}),$$

and by combining Eq. (3) and Eq. (4) with Eq. (2) we have

$$\mathbb{P}\left[X < Y_y\right] \leq \mathbb{P}\left[Y'_y \geq \alpha\right] + \mathbb{P}\left[X' \leq \alpha\right] = O(1/\sqrt{n}) + O(1/\sqrt{n}) = O(1/\sqrt{n}).$$

Hence the probability of event A is $O(1/\sqrt{n})$. By the union bound for events A and B, the probability of at least one of them occurring is $O(1/\sqrt{n}) + O(1/\sqrt{n}) = O(1/\sqrt{n})$. The desired result follows.

□

The following lemma states that when the root hits a leaf, the individual in the root is an offspring that came from a homogeneous leaf.

Lemma S2. *Let π be a random modified trajectory, and consider that at some time t_1 the i-th leaf is hit by the root. Let t_2 and t_3 be the random variables which indicate that leaf i hits some leaf j at times (t_2, t_3). The probability that (t_1, t_2, t_3) is a heterogeneous hit is $O(1/\sqrt{n})$.*

Proof Idea. Note that in order for leaf i to hit leaf j, the following two events need to occur in succession.

(A) Leaf i hits the root s, and afterwards

(B) the root s reproduces before it is hit.

Since there are n leaves, given event A, the probability of event B happening is $O(1/n)$, and thus, in expectation, event A will need to happen $\Omega(n)$ times before B happens. On the other hand, observing event A for $\Omega(n)$ times takes long enough so that leaf i has become homogeneous whp. $\square$

Proof. Note that in a random modified trajectory the root reproduces with rate no larger than the rate with which it reproduces in a random regular trajectory. Let Z be the random variable that counts the number of times leaf i hits the root in the interval $(t_1, t_2]$. We obtain a lower bound on Z by assuming that the root is the only mutant in the population after it is hit by leaf i (hence the root reproduces with highest possible probability), and π is a random regular trajectory. In particular, we have $\mathbb{P}[Z \leq \alpha] \leq \mathbb{P}[Z' \leq \alpha]$ for all α, where Z' is geometrically distributed with rate $(2 \cdot r)/n$, which is an upper bound on the probability that the root reproduces before it is hit again, i.e.

$$Z' \sim \mathsf{GM}(p_Z) \qquad \text{where} \qquad p_Z = \frac{2 \cdot r}{n}$$

Then

$$\mathbb{P}\left[Z \leq \sqrt{n}\right] \leq \mathbb{P}\left[Z' \leq \sqrt{n}\right] = 1 - (1 - p_Z)^{\sqrt{n}} = O(1/\sqrt{n}) \tag{5}$$

Hence, the probability to observe that (t_1, t_2, t_3) is a heterogeneous when leaf i is chosen for reproduction at most $\sqrt{n}$ times in the interval $(t_1, t_2]$ is $O(1/\sqrt{n})$. On the other hand, the probability to observe the desired event when leaf i is chosen for reproduction at least $\sqrt{n}$ times requires that the leaf has remained heterogeneous after at least $\sqrt{n}$ reproduction events have occurred locally. In turn, this event requires that leaf i is hit by the root before it becomes homogeneous, or it has remained heterogeneous after $\sqrt{n}$ reproduction events have occurred locally, given that it has not been hit by the root. By Lemma S1, the probability that leaf i is hit by the root before it becomes homogeneous is $O(1/\sqrt{n})$. Finally, since $m = O(1)$, if we condition on the fact that leaf i is not hit by the root before it becomes homogeneous, the probability that it has remained heterogeneous after it has been chosen for reproduction $\sqrt{n}$ times is $O(1/\sqrt{n})$. The desired result follows. $\square$

Lemma S3. *Let π be a random modified trajectory, any position t of π. The probability that the root reproduces after t before it is hit by a leaf is $O(1/n)$.*

Proof. Note that in a random modified trajectory the root reproduces with rate no larger than the rate with which it reproduces in a random regular trajectory. In a random regular trajectory, the root is chosen for reproduction with rate at most r/n, whereas it is hit by the leaves with rate at least ε, for some constant ε. Then the probability that the root is chosen for reproduction before it is hit by a leaf is at most

$$\frac{\frac{r}{n}}{\frac{r}{n} + \varepsilon} = O(1/n)$$

Hence the event in consideration occurs with probability $O(1/n)$. $\square$

Lemma S4. *Let π be a random modified trajectory, and t any position of π. Let t_1, t_2 be the random variables that indicate a hitting pair (t_1, t_2), with $t_1 > t$. Let $e_{t_1} = (a_{t_1}, b_{t_1})$ and $e_{t_2} = (a_{t_2}, b_{t_2})$. If a_{t_1} and b_{t_2} belong to homogeneous leaves of different types, then the probability that a_{t_1} belongs to a resident leaf is at most $1/(r^2 + 1) + O(1/n)$.*

Proof Idea. The proof is by showing that for any modified trajectory π^- up to time t_2, and in which a_1 belongs to a resident leaf, if $t_2 - t_1$ is "reasonably small", then there exists a modified trajectory π^+ up to time t_2 where a_{t_1} belongs to a mutant leaf and such that

$$\frac{\mathbb{P}[\pi^-]}{\mathbb{P}[\pi^+]} \leq \frac{1}{r^2} \cdot (1 + O(1/n))$$

The modified trajectory π^+ is obtained from π^- by a form of "mirroring". In particular, π^+ is identical to π^-, with a_{t_1} and b_{t_2} swapped, so that if leaf i hits leaf j at times (t_1, t_2) in π^-, then leaf j hits leaf i at times (t_1, t_2) in π^+. $\square$

Proof. Let $\pi^- = e_1^-, \dots, e_{t_2}^-$ be any modified trajectory up to time t_2 in which a_1 belongs to a resident leaf. Observe that in every round the root is hit with probability at least ε, for some constant $\varepsilon > 0$. Let $\gamma = -\log(1-\varepsilon)$. First we show that the probability that $t_2 - t_1 - 1 > (1/\gamma) \cdot \log n$ is $O(1/n)$. Indeed, since (t_1, t_2) is a hitting pair, the root is not hit in any e_i^- for $t_1 < i < t_2$, and the probability of this event happening is at most $(1-\varepsilon)^{t_2 - t_1 - 1}$. Then, for $t_2 - t_1 - 1 > (1/\gamma) \cdot \log n$, this event happens with probability at most

$$(1-\varepsilon)^{\frac{1}{\gamma}\cdot \log n} = \left(\frac{1}{1-\varepsilon}\right)^{-\frac{1}{\gamma}\cdot \log n} = 2^{-\gamma \cdot \frac{1}{\gamma} \cdot \log n} = 2^{-\log n} = O(1/n) \tag{6}$$

Now assume that $t_2 - t_1 - 1 \leq (1/\gamma) \cdot \log n$. The probability of π^- is

$$\mathbb{P}\left[\pi^-\right] = \prod_{i=1}^{t_2} \mathbb{P}\left[\mathcal{X}_i = e_i^- | \pi_i^-\right] = \left(\prod_{i=1}^{t_1} \mathbb{P}\left[\mathcal{X}_i = e_i^- | \pi_i^-\right]\right) \cdot \left(\prod_{i=t_1+1}^{t_2} \mathbb{P}\left[\mathcal{X}_i = e_i^- | \pi_i^-\right]\right)$$

We obtain a modified trajectory π^+ by replacing e_{t_1} and e_{t_2} with e'_{t_1} and e'_{t_2} respectively, where $e'_{t_1} = (b_{t_2}, a_{t_2})$ and $e'_{t_2} = (b_{t_1}, a_{t_1})$. Let $\pi^+ = e_1^+, \dots, e_{t_2}^+$, and then (t_1, t_2) is a hitting pair in π^+, in which the reproducing leaf $e_{t_1}^+$ is mutant.

We have

$$\mathbb{P}\left[\pi^+\right] = \prod_{i=1}^{k} \mathbb{P}\left[\mathcal{X}_i = e_i^+ | \pi_i^+\right] = \left(\prod_{i=1}^{t_1} \mathbb{P}\left[\mathcal{X}_i = e_i^+ | \pi_i^+\right]\right) \cdot \left(\prod_{i=t_1+1}^{t_2} \mathbb{P}\left[\mathcal{X}_i = e_i^+ | \pi_i^+\right]\right)$$

Since $\pi_{t_1}^+ = \pi_{t_1}^-$, we have

$$\prod_{i=1}^{t_1} \frac{\mathbb{P}\left[\mathcal{X}_i = e_i^- | \pi_i^-\right]}{\mathbb{P}\left[\mathcal{X}_i = e_i^+ | \pi_i^+\right]} = \frac{\mathbb{P}\left[\mathcal{X}_i = e_{t_1}^- | \pi_i^-\right]}{\mathbb{P}\left[\mathcal{X}_i = e_{t_1}^+ | \pi_i^+\right]} = \frac{\mathbb{P}\left[\mathcal{X}_i = e_{t_1} | \pi_{t_1}^-\right]}{\mathbb{P}\left[\mathcal{X}_i = e'_{t_1} | \pi_{t_1}^-\right]} = \frac{1}{r} \tag{7}$$

Let f_i^- and f_i^+ be the fitness of the population right before events e_i^- and e_i^+ occur, respectively. Since after position t_1 there is one more mutant in π^+ than π^-, we have $f_i^+ = f_i^- + r - 1$ for $i \geq t_1$. Since $f_i^- = \Omega(n)$, we have

$$\prod_{i=k'+1}^{k} \frac{f_i^+}{f_i^-} = \prod_{i=k'+1}^{k} \frac{(f_i^- + r - 1)}{f_i^-} \leq (1 + O(1/n))^{k-k'+1} \leq (1 + O(1/n))^{(1/\gamma)\cdot \log n + 1} \leq 1 + O(1/n)$$

thus

$$\prod_{i=k'+1}^{k} \frac{\mathbb{P}\left[\mathcal{X}_i = e_i^- | \pi_i^-\right]}{\mathbb{P}\left[\mathcal{X}_i = e_i^+ | \pi_i^+\right]} \leq \frac{1}{r} \cdot \prod_{i=k'+1}^{k} \frac{f_i^+}{f_i^-} \leq \frac{1}{r} \cdot (1 + O(1/n)) \tag{8}$$

Using Eq. (7) and Eq. (8) we obtain

$$\frac{\mathbb{P}\left[\pi^-\right]}{\mathbb{P}\left[\pi^+\right]} = \left(\prod_{i=1}^{t_1} \frac{\mathbb{P}\left[\mathcal{X}_i = e_i^- | \pi_i^-\right]}{\mathbb{P}\left[\mathcal{X}_i = e_i^+ | \pi_i^+\right]}\right) \cdot \left(\prod_{i=t_1+1}^{t_2} \frac{\mathbb{P}\left[\mathcal{X}_i = e_i^- | \pi_i^-\right]}{\mathbb{P}\left[\mathcal{X}_i = e_i^+ | \pi_i^+\right]}\right) \leq \frac{1}{r} \cdot \frac{1}{r} \cdot (1 + O(1/n)) = \frac{1}{r^2} \cdot (1 + O(1/n)) \tag{9}$$

Overall, the probability that a_{t_1} belongs to a resident leaf is bounded by the sum of the probabilities of this event happening when (i) $t_2 - t_1 - 1 > (1/\gamma) \cdot \log n + 1$ and (ii) $t_2 - t_1 - 1 \leq (1/\gamma) \cdot \log n + 1$. By Eq. (6), case (i) happens with probability $O(1/n)$, whereas by Eq. (9) case (ii) happens with probability

$$\frac{1}{1 + \frac{r^2}{1 + O(1/n)}} \leq \frac{1}{r^2 + 1} + O(1/n)$$

Thus the event under consideration happens with probability at most

$$O(1/n) + \frac{1}{r^2+1} + O(1/n) = \frac{1}{r^2+1} + O(1/n)$$

The desired result follows. □

2.3 A Coupling Argument for the Fixation Probability on $\mathsf{M}_{N(n)}^{G_m}$

In this section we introduce a simple Markov chain $\mathcal{M}_n$ and use a coupling argument to argue that the fixation probability on the Metastar is underapproximated by the probability that a random walk on $\mathcal{M}_n$ gets absorbed in a particular state.

To simplify notation, we let $\alpha = \rho^-(r, G_m, v)$ and $\beta = \rho^+(r, G_m, v)$. We define a Markov chain $\mathcal{M}_n = (S, \delta)$ which consists of the set of states S:

1. s_i, for $0 \leq i \leq n$,
2. η_i, for $0 < i < n$,
3. θ_i, for $0 < i < n$.

Let $z = c/\sqrt{n}$ where c is a large enough constant. The transition probability function $\delta : S \times S \to [0,1]$ is defined such that for all $0 < i < n$ we have:

1. $\delta(s_i, \eta_i) = \frac{r^2}{r^2+1} - 2 \cdot z$,
2. $\delta(s_i, \theta_i) = \frac{1}{r^2+1} + z$,
3. $\delta(s_i, s_{i-1}) = z$,
4. $\delta(\eta_i, s_{i+1}) = \beta - z$,
5. $\delta(\eta_i, s_i) = 1 - \beta + z$,
6. $\delta(\theta_i, s_{i-1}) = \alpha + z$,
7. $\delta(\theta_i, s_i) = 1 - \alpha - z$,

whereas $\delta(s_0, s_0) = \delta(s_n, s_n) = 1$ (i.e., the states s_0 and s_n are absorbing).

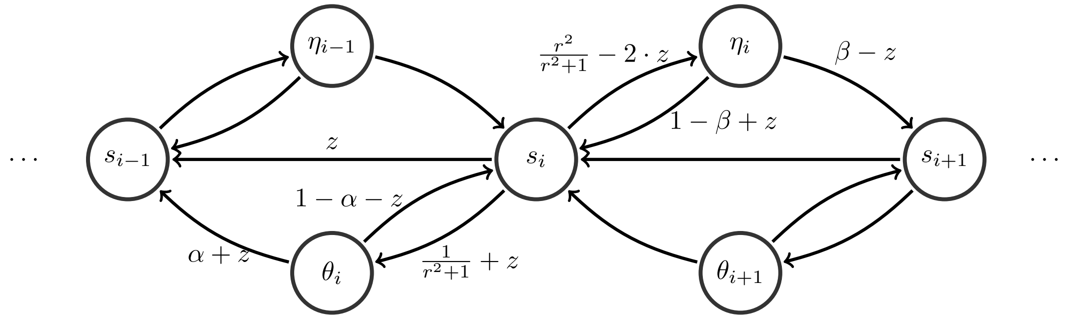

Figure S1: The Markov chain $\mathcal{M}_n$.

Intuition. Intuitively, a random walk on $\mathcal{M}_n$ starting from state s_1 models the modified Moran process on $\mathsf{M}_{N(n)}^{G_m}$ starting from a mutant leaf. Whenever the random walk is on some state s_i, there are at most $n - i$ resident leaves in $\mathsf{M}_{N(n)}^{G_m}$. A transition to state η corresponds to the event of a mutant leaf hitting a resident leaf. A transition to state θ corresponds to the event of a resident leaf hitting a mutant leaf.

Lemma S5. *Consider that at some time t^* the modified Moran process on $\mathsf{M}^{G_m}_{N(n)}$ reaches a configuration S_{t^*} which contains a mutant leaf. Then the fixation probability on $\mathsf{M}^{G_m}_{N(n)}$ is at least the probability that a random walk on $\mathcal{M}_n$ starting from state s_0 eventually gets absorbed in state s_n.*

Proof Idea. Let ψ be a fixed trajectory of the modified Moran process of to time t^*, which generates the desired configuration S_{t^*}. Let Π_1 be the modified Moran process on $\mathsf{M}^{G_m}_{N(n)}$ starting from S_t. Similarly, let Π_2 be the random process on $\mathcal{M}_n$ starting from s_1. We couple Π_1 and Π_2, so that whenever Π_2 is on state s_i of $\mathcal{M}_n$, there are at most $n-i$ (homogeneous) resident leaves of $\mathsf{M}^{G_m}_{N(n)}$ in Π_1. We do so by first extending ψ indefinitely, and then using ψ with some biased coins as the source of randomness for Π_2. The correctness of the coupling relies on the following.

1. Lemma S3, which guarantees that when the root hits a leaf j, it has previously been hit by some leaf i, and hence leaf i hits leaf j.
2. Lemma S2, which guarantees that leaves i and j are homogeneous whp.
3. Lemma S4, which captures the forward bias of leaves i and j being a mutant leaf and resident leaf, respectively.
4. Lemma S1, which guarantees that after being hit, leaf j resolves to a homogeneous state independently, whp.

□

Proof. We describe the process of associating certain events in ψ with events in Π_2. In this process, we will be erasing some events e_i of ψ where the reproducing individual of e_1 occupies the root of $\mathsf{M}^{G_m}_{N(n)}$. Thus we will be working in general with a modified trajectory π. Initially, π is identical to ψ.

Whenever Π_2 transitions to some state η_i or θ_i, this will correspond to some leaf j of $\mathsf{M}^{G_m}_{N(n)}$ being hit by the root in π. Then we will refer to leaf j as the *active leaf* while in η_i or θ_i. In particular, an active leaf in η_i is one that is hit with a mutant and was a resident leaf in the last round, whereas an active leaf in θ_i is one that is hit with a resident, and was a homogeneous mutant leaf in the last round.

1. Starting from s_1, we scan π from left to right until we find the first occurrence in π where some leaf j becomes heterogeneous. Let $\mathcal{E}_1$ be the event that the root reproduces before it is hit, and by Lemma S3, we have $\mathbb{P}[\mathcal{E}_1] \leq z$. If $\mathcal{E}_1$ does not hold, we flip a coin with probability of heads $z - \mathbb{P}[e_1]$, and let $\mathcal{E}_2$ be the event that the coin comes up heads. We make Π_2 take the transition $s_1 \to s_0$ if either $\mathcal{E}_1$ or $\mathcal{E}_2$ hold. Note that the second event is conditioned on the failure of the first (i.e., we assign $\mathbb{P}[\mathcal{E}_2|\mathcal{E}_1] = 0$), thus $\mathcal{E}_1$ and $\mathcal{E}_2$ are disjoint, and hence their union occurs with probability
$$\mathbb{P}[\mathcal{E}_1] + \mathbb{P}[\mathcal{E}_2] = z$$
Now assume that both $\mathcal{E}_1$ and $\mathcal{E}_2$ fail. Since $\mathcal{E}_1$ does not occur, the leaf j is turned heterogeneous by a hitting pair (t, t'), and let $e_t = (a_t, b_t), e_{t'} = (a_{t'}, b_{t'})$. Let $\mathcal{E}_3$ be the event that a_t belongs to a resident leaf, and by Lemma S4,
$$\mathbb{P}[\mathcal{E}_3] \leq \frac{1}{r^2+1} + z$$
If $\mathcal{E}_3$ does not hold, we flip a coin with probability of heads $z - \mathbb{P}[\mathcal{E}_3]$, and let $\mathcal{E}_4$ be the event that the coin comes up heads. We make Π_2 take the transition $s_1 \to \theta_1$ if either $\mathcal{E}_3$ or $\mathcal{E}_4$ occurs. Additionally, we mark leaf j as the active leaf in θ_1. Finally, we make Π_2 take the transition $s_1 \to \eta_1$ if none other transition has been taken, i.e. with probability
$$1 - \left(\frac{1}{r^2+1} + z\right) = \frac{r^2}{r^2+1} - z$$
and mark leaf j as the active leaf in η_1. Additionally, we erase event $e_{t'}$ from π.
2. While in state η_i with active leaf j, let $\mathcal{E}_1$ be the event that leaf j is hit by the root at some time t before it becomes homogeneous, and by Lemma S1, we have $\mathbb{P}[\mathcal{E}_1] \leq z$. If $\mathcal{E}_1$ occurs, we erase the event e_t from π. If $\mathcal{E}_1$ does not occur, let $\mathcal{E}_2$ be the event that the j-th leaf becomes a resident leaf the next time it becomes homogeneous. Since this leaf has been hit with a mutant, we have $\mathbb{P}[\mathcal{E}_2] = 1 - \beta$. Finally, if neither $\mathcal{E}_1$ nor $\mathcal{E}_2$

hold, we flip a coin with probability of heads $z-\mathbb{P}[\mathcal{E}_1]$, and let $\mathcal{E}_3$ be the event that the coin comes up heads. We make Π_2 take the transition $\eta_i \to s_i$ if any of the events $\mathcal{E}_1$, $\mathcal{E}_2$ and $\mathcal{E}_3$ occurs, which happens with probability

$$\mathbb{P}[\mathcal{E}_1] + \mathbb{P}[\mathcal{E}_2] + \mathbb{P}[\mathcal{E}_3] = 1 - \beta + z$$

and make Π_2 take the transition $\eta_i \to s_{i+1}$ otherwise, i.e. with probability $\beta - z$.

3. While in state θ_i with active leaf j, let $\mathcal{E}_1$ be the event that leaf j is hit by the root at some time t before it becomes heterogeneous, and by Lemma S1, we have $\mathbb{P}[\mathcal{E}_1] \leq z$. If $\mathcal{E}_1$ does not occur, let $\mathcal{E}_2$ be the event that the j-th leaf becomes a resident leaf the next time it becomes homogeneous. Since this leaf has been hit with a resident and was a homogeneous mutant leaf before, we have $\mathbb{P}[\mathcal{E}_2] = \alpha$. Finally, if neither $\mathcal{E}_1$ nor $\mathcal{E}_2$ hold, we flip a coin with probability of heads $z - \mathbb{P}[\mathcal{E}_1]$, and let $\mathcal{E}_3$ be the event that the coin comes up heads. We make Π_2 take the transition $\theta_i \to s_{i-1}$ if any of the events $\mathcal{E}_1$, $\mathcal{E}_2$ and $\mathcal{E}_3$ occurs, which happens with probability

$$\mathbb{P}[\mathcal{E}_1] + \mathbb{P}[\mathcal{E}_2] + \mathbb{P}[\mathcal{E}_3] = \alpha + z$$

and make Π_2 take the transition $\theta_i \to s_i$ otherwise, i.e. with probability $1 - \alpha - z$.

4. While in state s_i, let j be the last active leaf (i.e., the leaf that was active the last time Π_2 was in either a state $\eta_{i'}$ or $\theta_{i'}$), and t_1 the position in π that turned leaf j heterogeneous. Let t_2 be the first time after t_1 such that π has a hitting pair (t_2, t_3), and $\mathcal{E}_1$ the event that (t_1, t_2, t_3) constitutes a heterogeneous hit. By Lemma S2, we have $\mathbb{P}[\mathcal{E}_1] \leq z$. If $\mathcal{E}_1$ occurs, we erase from π the event e_{t_3}. If $\mathcal{E}_1$ does not occur, let $\mathcal{E}_2$ be the event that the root reproduces at some time $t_4 > t_1$ and it is not hit in the interval (t_1, t_4). By Lemma S3, we have $\mathbb{P}[\mathcal{E}_2] \leq z$. If $\mathcal{E}_2$ occurs, we erase from π the event e_{t_4}. If neither $\mathcal{E}_1$ nor $\mathcal{E}_2$ occurs, we flip a coin with probability of heads $z - \mathbb{P}[\mathcal{E}_1] - \mathbb{P}[\mathcal{E}_2]$, and let $\mathcal{E}_3$ be the event that the coin comes up heads. We make Π_2 take the transition $s_i \to s_{i-1}$ if either $\mathcal{E}_1$ or $\mathcal{E}_2$ occurs, which happens with probability

$$\mathbb{P}[\mathcal{E}_1] + \mathbb{P}[\mathcal{E}_2] + \mathbb{P}[\mathcal{E}_3] = z$$

If none of $\mathcal{E}_1$, $\mathcal{E}_2$ and $\mathcal{E}_3$ occur, we scan π to the right from position t_1, and find the next hitting pair (t, t'), and let $e_t = (a_t, b_t), e_{t'} = (a_{t'}, b_{t'})$. Note that a_t and $b_{t'}$ necessarily belong to homogeneous leaves. Let $\mathcal{E}_4$ be the event that a_t belongs to a resident leaf, and by Lemma S4 we have

$$\mathbb{P}[\mathcal{E}_4] \leq \frac{1}{r^2+1} + z$$

If $\mathcal{E}_4$ does not occur, we flip a coin with probability of heads $z-\mathbb{P}[\mathcal{E}_4]$, and let $\mathcal{E}_5$ be the event that the coin comes up heads. We make Π_2 take the transition $s_i \to \theta_i$ if either $\mathcal{E}_4$ or $\mathcal{E}_5$ occurs, which happens with probability

$$\mathbb{P}[\mathcal{E}_4] + \mathbb{P}[\mathcal{E}_5] = \frac{1}{r^2+1} + z$$

Additionally, we mark leaf j as the active leaf in θ_i. Finally, we make Π_2 take the transition $s_i \to \eta_i$ if none other transition has been taken, i.e. with probability

$$1 - \left(\frac{1}{r^2+1} + z + z\right) = \frac{r^2}{r^2+1} - 2 \cdot z$$

and mark leaf j as the active leaf in η_i. Additionally, we erase event $e_{t'}$ from π.

The desired result follows directly from the coupling process. □

The following lemma establishes the forward bias on the Markov chain $\mathcal{M}_n$, i.e. given a current state s_i, the ratio of the probabilities of transitioning to state s_{i+1} over transitioning to state s_{i-1}.

Lemma S6. *For any $0 < i < n$, let x_{s_i} be the probability that a random walk on $\mathcal{M}_n$ starting from state s_i transitions to state s_{i+1} before it transitions to state s_{i-1}. We have*

$$\frac{x_{s_i}}{1 - x_{s_i}} = \frac{\frac{r^2}{r^2+1} \cdot \beta - z \cdot \left(\frac{r^2}{r^2+1} + \beta - z\right)}{\frac{1}{r^2+1} \cdot \alpha + z \cdot \frac{r^2+2}{r^2+1}}$$

Proof. Given that a random walk is in state s_i (resp. η_i, β_i), $0 < i < n$, let x_{s_i} (resp. x_{η_i}, x_{β_i}) be the probability that the walk transitions to s_{i+1} before it transitions to s_{i-1}. Then we have the following system

$$\left.\begin{aligned} x_{s_i} &= (\tfrac{r^2}{r^2+1} - z) \cdot x_{\eta_i} + \tfrac{1}{r^2+1} \cdot x_{\beta_i} \\ x_{\eta_i} &= \beta - z + (1-\beta+z) \cdot x_{s_i} \\ x_{\beta_i} &= (1-\alpha-z) \cdot x_{s_i} \end{aligned}\right\} \implies x_{s_i} = \frac{\left(\frac{r^2}{r^2+1} - z\right) \cdot (\beta - z)}{1 - \left(\frac{r^2}{r^+1} - z\right) \cdot (1-\beta+z) - \frac{1}{r^2+1} \cdot (1-\alpha-z)}$$

$$\implies \frac{x_{s_i}}{1 - x_{s_i}} = \frac{\frac{r^2}{r^2+1} \cdot \beta - z \cdot \left(\frac{r^2}{r^2+1} + \beta - z\right)}{\frac{1}{r^2+1} \cdot \alpha + z \cdot \frac{r^2+2}{r^2+1}}$$

The desired result follows. □

The following theorem captures the fixation probability on the metastar family.

Theorem S1. *Let G_m be a fixed graph and v the attachment vertex of G_m. Denote $p = \rho(r, G_m, v)$ and $\alpha = \rho^-(r, G_m, v)$ and $\beta = \rho^+(r, G_m, v)$. The fixation probability of a single mutant placed uniformly at random on $\mathsf{M}^{G_m}_{N(n)}$ is*

$$\rho\left(r, \mathsf{M}^{G_m}_{N(n)}\right) \geq p \cdot \frac{1 - r^{-2} \cdot (\alpha/\beta)}{1 - (r^{-2} \cdot (\alpha/\beta))^n} \cdot (1 + o(1)) \tag{10}$$

Proof. First, note that a mutant placed uniformly at random on $\mathsf{M}^{G_m}_{N(n)}$ will be placed in a leaf with probability $1 - O(1/n)$. Then $\rho\left(r, \mathsf{M}^{G_m}_{N(n)}\right)$ is lowerbounded by the probability of that mutant fixating in the initial leaf, times the probability that the mutants fixate in $\mathsf{M}^{G_m}_{N(n)}$ starting from a mutant leaf. The former event occurs with probability $p = \rho(r, G_m, v)$. By Lemma S5, the probability of the latter event is lowerbounded by the probability ϕ that a random walk on $\mathcal{M}_n$ starting from s_1 will result in s_n. Let x_{s_i} be the probability that a random walk on $\mathcal{M}_n$ starting from state s_i transitions to state s_{i+1} before it transitions to state s_{i-1}, and $\gamma = \frac{x_{s_i}}{1 - x_{s_i}}$. Using Lemma S6, the probability that a random walk of $\mathcal{M}_n$ from s_1 will get absorbed in s_n is

$$\phi = \frac{1}{\sum_{i=0}^{n-1} \left(\frac{1 - x_{s_i}}{x_{s_i}}\right)^i} = \frac{1}{\sum_{i=0}^{n-1} \gamma^{-i}} = \frac{1 - \gamma^{-1}}{1 - \gamma^{-n}} = \frac{1 - r^{-2} \cdot (\alpha/\beta)}{1 - (r^{-2} \cdot (\alpha/\beta))^n} \cdot (1 + o(1))$$

The desired result follows. □

References

[1] Martin A Nowak. *Evolutionary dynamics : exploring the equations of life*. Belknap Press of Harvard University Press, Cambridge, Massachusetts, 2006.
